# Comparison of the Canadian CT head rule and the new orleans criteria in patients with minor head injury

**DOI:** 10.1186/1749-7922-9-31

**Published:** 2014-04-17

**Authors:** Cemil Kavalci, Gokhan Aksel, Omer Salt, M Serkan Yilmaz, Ali Demir, Gulsüm Kavalci, Betul Akbuga Ozel, Ertugrul Altinbilek, Tamer Durdu, Cihat Yel, Polat Durukan, Bahattin Isik

**Affiliations:** 1 Emergency Department, Baskent University Faculty of Medicine, Ankara, Turkey; 2 Emergency Department, State hospital, Yozgat, Turkey; 3 Emergency Department, Numune Training and Research Hospital, Ankara, Turkey; 4 Emergency Department, Yenimahalle State hospital, Ankara, Turkey; 5 Anesthesia Department, Yenimahalle State hospital, Ankara, Turkey; 6 Emergency Department, Şİşli Hamidiye Etfal Training and Research Hospital, İstanbul, Turkey; 7 Emergency Department, Erciyes University Faculty of Medicine, Kayseri, Turkey; 8 Emergency Department, Keciören Training and Research Hospital, Ankara, Turkey

**Keywords:** Emergency, Head injury, CT rules

## Abstract

**Aim:**

The aim of the study was to compare the New Orleans Criteria and the New Orleans Criteria according to their diagnostic performance in patients with mild head injury.

**Methods:**

The study was designed and conducted prospectively after obtaining ethics committee approval. Data was collected prospectively for patients presenting to the ED with Minor Head Injury. After clinical assessment, a standard CT scan of the head was performed in patients having at least one of the risk factors stated in one of the two clinical decision rules.

Patients with positive traumatic head injury according to BT results defined as Group 1 and those who had no intracranial injury defined as Group 2. Statistical analysis was performed with SPSS 11.00 for Windows. ROC analyze was performed to determine the effectiveness of detecting intracranial injury with both decision rules. p < 0.05 was considered statistically significant.

**Results:**

175 patients enrolled the study. Male to female ratio was 1.5. The mean age of the patients was 45 ± 21,3 in group 1 and 49 ± 20,6 in group 2. The most common mechanism of trauma was falling. The sensitivity and specificity of CCHR were respectively 76.4% and 41.7%, whereas sensitivity and specificity of NOC were 88.2% and 6.9%.

**Conclusion:**

The CCHR has higher specificity, PPV and NPV for important clinical outcomes than does the NOC.

## Introduction

Minor head injury (MHI) is one of the most common injury type seen in the emergency departments (ED) [1]. The average incidence of MHI is reported to be 503.1/100000, with peaks among males and those <5 years of age [2]. No universally agreed definition of MHI exists. Some authors define MHI as the blunt injury of the head with alteration in consciousness, amnesia, or disorientation in a patient who has a Glasgow Coma Scale (GCS) score of 13 to 15 [3,4], although others define it as the blunt injury of the head with alteration in consciousness, amnesia, or disorientation in a patient who has a Glasgow Coma Scale (GCS) score of 14 to 15 [5]. The key to managing these patients is early diagnosis of intracranial injuries using computed tomography (CT) [6,7]. CT is widely accepted as an effective diagnostic modality to detect rare but clinically significant intracranial injuries in patients suffering minor head injury [8]. As such, it has been increasingly utilized as a routine test for these patients [9]. Systematic evaluation by CT scan would not be a cost-effective strategy in mild head injury because potentially life-threatening complications that may require neurosurgical intervention occur in less than 1% of cases [4]. In addition, some reports warn against its harmful effects (particularly for children) due to the radiation exposure [10]. Yet, CT use is growing rapidly, potentially exposing patients to unnecessary ionizing radiation risk and costs [11].

Commonly accepted clinical decision rules for detecting life-threatening complications in patients with mild head injury are New Orleans Criteria (NOC) and the Canadian CT Head Rules (CCHR) [3,4,12]. These two rules were externally validated in the previous studies but we believe that application of these decision rules may still be limited in populations with different demographic and epidemiologic features. The aim of the study was to compare the CCHR and the NOC according to their diagnostic performance in MHI patients.

## Materials and methods

This study was conducted at a single tertiary care center in Turkey with an annual ED census of 70,000 visits. The study was designed and conducted prospectively after ethics committee approval. Acute MHI was defined as a patient having a blunt trauma to the head within 24 hours, with a Glasgow Coma Scale (GCS) score of 13 to 15. The patients were also required to have at least one of the risk factors stated in CCHR or NOC (Table 1). Patients with GCS score of less than 13 or instable vital signs, presentation time more than 24 hours after head trauma, patients with an obvious penetrating skull injury or obvious depressed fracture, presence of major trauma, bleeding disorder or use of oral anticoagulants (e.g., warfarin), contraindications for CT and those pregnant or fewer than 18 were excluded from the study.

**Table 1:** Canadian CT head rule and New Orleans Criteria

**Canadian CT Head Rule High risk (for neurosurgical interventions)**	**New Orleans Criteria**
• GCS score, 15 at two hours after injury	• Headache
• Suspected open or depressed skull fracture	• Vomiting
• Any sign of basal skull fracture (hemotympanum, “panda” eyes, cerebrospinal fluid otorrhoea, Battle’s sign).	• Older than 60 years
• Vomiting more than once	• Drug or alcohol intoxication
• Age >65 years	• Persistent anterograde amnesia (deficits in short-term memory)
Medium risk (for brain injury on CT)	
• Persistent retrograde amnesia of greater than 30 minutes	• Visible trauma above the clavicle
• Dangerous mechanism of injury (pedestrian struck by vehicle, ejection from vehicle, fall from greater than three feet or five stairs)	• Seizure

All patients were assessed by an emergency physician or by supervised emergency medicine residents. Data collection was done prospectively using a data collection sheet. After clinical assessment, a standard CT scan of the head was performed in patients having at least one of the risk factors stated in one of the two clinical decision rules. The CT scans were interpreted by a radiologist who was blinded to patient data. Presence of traumatic lesions on head CT scan was the main outcome. The lesions accepted as positive CT results for the study were subarachnoid hemorrhage, epidural hemorrhage, subdural hematoma, intraparenchymal hematoma, compression fracture, cerebral edema and contusion.

Cases without a complete data sheet were excluded. Demographic characteristics, mechanism of injury, traumatic findings at CT were all evaluated. CCHR and NOC were also assessed in patients who presented with a minor head trauma. Patients with positive traumatic head injury according to BT results defined as Group 1 and those who had no intracranial injury defined as Group 2. Statistical analysis was performed with SPSS (version 11.0; SPSS, Inc., Chicago, IL). Results were expressed with number and percentage. Chi-square test was used in comparison of categorical data. ROC analyze was performed to determine the effectiveness of detecting intracranial injury with both decision rules. The sensitivity, specificity, and predictive values with 95% confidence intervals (CIs) for performance of each decision rule for CT scan intracranial traumatic findings were calculated separately for patients having GCS score of 13 and patients having GCS score of 14–15. P < 0.05 was considered statistically significant. When appropriate, CIs were calculated with a 95% confidence level.

## Results

During the study period, data were collected for 198 trauma patients who met inclusion criteria. Of these, 21 were excluded because of refusing to be included in the study, 2 were excluded because of missing data, resulting in 175 patients in the data analysis. Table 2 shows the demographic and clinical characteristics of the overall study group. In the enrolled patients, male to female ratio was 1.5. The mean age of the patients was 45 ± 21.3 in group 1 and 49 ± 20.6 in group 2. The most common mechanism of trauma was falling. Headache was the main symptom in both groups (Table 2). CT scan was performed in all of 175 patients; pathologic findings were present in 17 patients (9.71%). The most common intracranial injury was Subarachnoid hemorrhage (Table 3).

**Table 2:** Characteristics of patients

	**Group 1**	**Group 2**	**P value**
Sex (male/female)	14/3	92/66	p>0,05
Age (mean ± sd*)	45 ± 21,3	49.57 ± 20,6	p>0,05
Trauma mechanism			
Motor vehicle accident	2	34	
Pedestrian	0	8	p>0,05
Falling	8	68	
Assault	7	48	
Symptom			
Headache	12	139	
Amnesia	1	7	
Vomiting	2	19	
Lethargy	3	6	
Loss of consciousness	1	9	
GCS			
13	3	4	
14	0	9	
15	14	145	

*Sd=standart deviation, GCS=Glasgow Coma Scale Score.

**Table 3:** Computed tomography results of the patients

**BT results**	**N**	**%**
Normal	156	89.1
Epidural hemorrhage	3	1.8
Depressed fracture	2	1.2
Cerebral edema	4	2.4
Subdural hematoma	3	1.8
Intraparenchymal hematoma	1	0.6
Subarachnoid hemorrhage	6	3.4
Contusion	2	1.2

**Figure 1:**
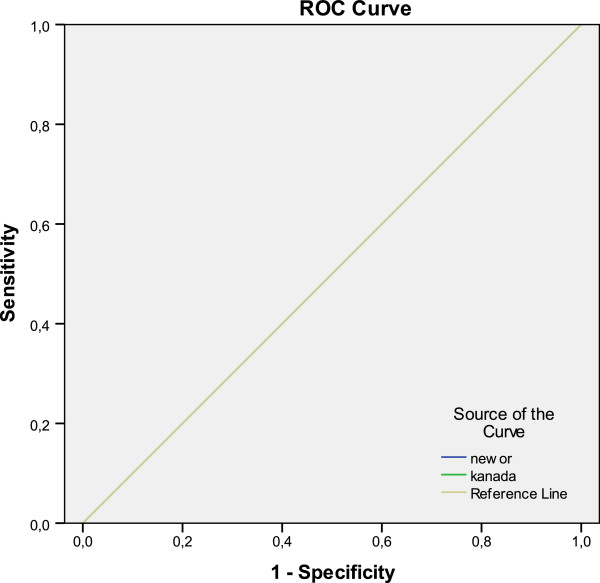
**Ratio of detecting intracranial injury of decision rules for patients with GCS 13.** Diagonal segments are produced by ties.

Sensitivity, Specificity, PPV, and NPV of both of the criteria of the patients having GCS score 13 were 100%, %0, 42% and 100% respectively (Table 4, Figure 1).

**Table 4:** Rates of patients meet the criteria according to groups for patients with GCS 13

**Predictor**	**Group 1**	**Group 2**
Canadian CT* Head Rule		
Positive	3	0
Negative	4	0
New Orleans Criteria		
Positive	3	0
Negative	4	0

**Figure 2:**
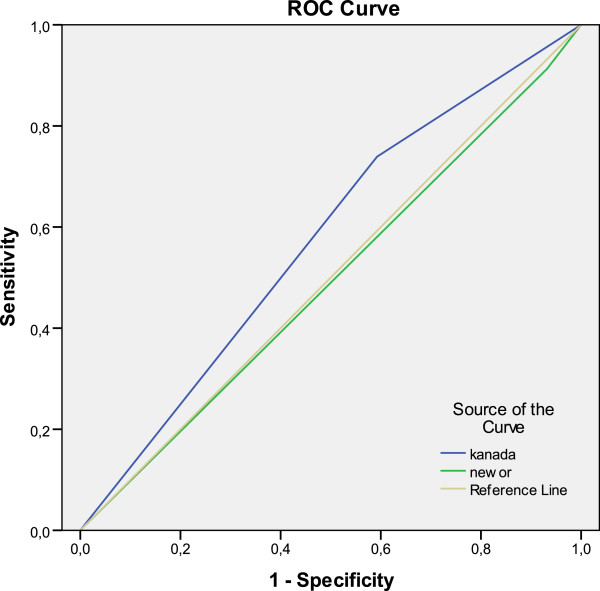
**Ratio of detecting intracranial injury of decision rules for patients with GCS 14-15.** Diagonal segments are produced by ties.

For the patients having GCS score between 14–15; the sensitivity and specificity of CCHR were 78.5% and 42.8% respectively, whereas sensitivity and specificity of NOC were 85.7% and 0.7%. Positive predictive value (PPV) and negative predictive value (NPV) were both higher in CCHR than NOC. PPV and NPV of CCHR were respectively 11.1% and 95.6% whereas PPV and NPV of NOC were 0.7% and 84.6% (Table 5, Figure 2).

**Table 5:** Rates of patients meet the criteria according to groups for patients with GCS 14-15

**Predictor**	**Group 1**	**Group 2**
Canadian CT* Head Rule		
Positive	11	88
Negative	3	66
New Orleans Criteria		
Positive	12	143
Negative	2	11

*CT= Computed tomography.

## Discussion

In the most of the prior studies, motor vehicle accidents were reported to be the most common mechanism of trauma [3,4]. Some other authors also reported the falling as the most common trauma mechanism [13]. In the present study, the most common mechanism for trauma was found as falling in accordance with the later study. Assault was the second and motor vehicle accidents were the third most common mechanisms of trauma. Our hospital is in the center of the city, and away from the high ways. This may be the reason for motor vehicle accidents to be the third most common cause. The mechanism of trauma is probably depends on the distance from hospital to high ways, social and economical status and degree or level of hospital as trauma centre. Similar to prior studies, males were the most affected sex group from the trauma in the present study [3,4,13]. This is probably due to men’s working in more dangerous jobs, taking more places in active city social life, being more associated with violence and male drivers being more than females.

In the present study, efficacy of both criteria were found similar in the patients having GCS score 13. In the patients having GCS score 14–15, a comparison of the clinical decision rules for use of CT in patients with MHI showed that both the CCHR and the NOC were sensitive for the outcome measure of any traumatic intracranial lesion on CT which is “clinically important” brain lesion. Although the sensitivity was high in these two decision rules, they both had much lower sensitivities in this study than the original published studies [3,13-15]. Papa et al. and Smits et al. found sensitivities of both rules to reach 100% [13,15]. The cause of lower sensitivities may be explained by our patients’ low socioeconomic status and unreliable history. In contrast to previous publications, Ro et al. found lower sensitivities in both decision rules similar to our study results. They also found the sensitivity higher in NOC and specificity higher in CCHR [16]. In the present study, the specificity of CCHR was higher than specificity of NOC (47,1% versus 6.9%). Our results were similar to the results of the study reported by Smits et al. They found the specificity of CCHR higher than the specificity of NOC (39.7% versus 5.6%) [13]. Papa et al. and Stiell et al. also found the specificity of CCHR higher than NOC [3,15]. In the present study, CCHR was found to be superior to NOC due to higher specificity, higher PPV and NPV. The only superiority of NOC in our study was the sensitivity with 88.2% while it was 76.4% in CCHR. Many prior studies also found the sensitivity of NOC higher than the sensitivity of CCHR [13,16]. Smits et al. tried to explain this difference in sensitivities for neurocranial traumatic CT findings between the 2 decision rules with more stringent use of the risk factor of external injury in the CCHR. For example in the NOC, this risk factor comprises all external injuries above the clavicles. Despite the NOC having higher sensitivity, specificities for neurocranial traumatic CT findings were low for the NOC decision rule, and higher for the CCHR [13]. In accordance with Smits et al. higher sensitivity of NOC causes the lower specificity and this means an increase in healthcare costs.

## Conclusion

In summary, for patients with MHI, the CCHR and the NOC have both high sensitivities for clinically important brain injury although this study reports much lower sensitivities than the prior published studies. Additionally, the CCHR has higher specificity, PPV and NPV for important clinical outcomes than does the NOC. We believe that use of CCHR may result in reduced imaging rates, reduced costs and this would help us to protect our patients from the side effects of radiation.

### Limitations

This study is conducted in one center. A multicenter study having larger number of patients and more trauma patients caused by much different mechanism could have been assessed. The study focused only on the two widely accepted clinical decision rules but did not study on other decision rules or aspects.

Our primary outcome measure was any traumatic neurocranial lesions on the CT scan. The third limitation of this study is absence of the second outcome measure which can be defined as findings on the CT scan that led to neurosurgical intervention.

## Competing interests

The authors declare that they have no competing interests.

## Authors’ contributions

The quantitative analysis was planned by CK, MSY, AD. Study data were analyzed by CK,CY,GK and interpreted by BAO, TD, EA, PD. The first version of the manuscript was drafted by AD, GA, BI. All authors contributed to the edition and revision of the manuscript and the final version of the article was reviewed and approved by all authors.
